# Visuomotor information drives interference between the hands more than dynamic motor information during bimanual reaching

**DOI:** 10.1007/s00221-026-07270-5

**Published:** 2026-03-16

**Authors:** Phillip C. Desrochers, Alexander T. Brunfeldt, Florian A. Kagerer

**Affiliations:** 1 Department of Kinesiology, Michigan State University, East Lansing, MI 48824 USA; 2 Neuroscience Program, Michigan State University, 308 W Circle Dr, East Lansing, MI 48824 USA

**Keywords:** Bimanual interference, Visuomotor perturbation, Dynamic perturbation, Neuromotor control

## Abstract

During complex bimanual movements, interference can occur in the form of one hand influencing the action of the contralateral hand. Interference likely results from conflicting sensorimotor information shared between brain regions controlling hand movements via neural crosstalk. However, how visual and force-related feedback processes interact with each other during bimanual reaching is not well understood. In this study, four groups experienced either a visuomotor perturbation, dynamic perturbation, combined visuomotor and dynamic perturbation, or no perturbation in their right hand during bimanual reaches, with each hand controlling its own cursor. The left hand was examined for interference as a consequence of the right-hand perturbation. The results indicated that the visuomotor and combined perturbations showed greater interference in the left hand than the dynamic perturbation. However, the combined perturbation did not show greater interference than the visuomotor perturbation. This suggests that dynamic sensorimotor and visuomotor processes do not interact between hemisphere-hand systems, and that primarily visuomotor processes lead to interference between the hands.

## Introduction

For humans, bimanual actions comprise a significant part of everyday life. While the adult neuromotor system is usually capable of performing simple or well-practiced bimanual tasks without problems (e.g., clapping, tying one’s shoes, typing), more complicated movements, particularly those with spatiotemporal incongruencies, can produce interference between the hands (e.g., the familiar childhood trick of rubbing one’s belly while patting one’s head, juggling, or playing the drums; Franz et al. 1991). In that context, interference is a phenomenon where the action of one hand can influence the action of the other hand, in the spatial and/or temporal domains. Interference during bimanual coordination has been well studied (Semjen et al. 1995; Kennerley et al. 2002; Hazeltine et al. 2003; Obhi and Goodale 2005; Kagerer 2016; Shea et al. 2016; Desrochers et al. 2020; Aune et al. 2021; Iester et al. 2025), and is thought to result from neural crosstalk between brain areas controlling bimanual movements. However, the specific type of sensorimotor information (i.e., direction, force etc.) being shared between the hemispheres, and how this information might interact in the context of interference, remains an open question.

It is possible to probe the function of different types of sensorimotor information within the CNS by asking participants to adapt to perturbations which manipulate specific sensorimotor components. To probe visuomotor processes, the visual feedback received by a participant can be manipulated to introduce a discrepancy between the intended and visually observed movement consequences. To probe dynamic motor processes, the forces imparted on the limbs during a movement can be manipulated to likewise provide unexpected dynamic feedback. Such perturbations require participants to modify so-called internal models (i.e., neural representations of the external world) and sensorimotor feedback gains to minimize the movement error introduced by the perturbation across successive trials (Wolpert et al. 1995; Kawato 1999; Körding and Wolpert 2004; Izawa and Shadmehr 2008; Franklin et al. 2012). When these perturbations are applied to one hand during bimanual reaches, spatial interference in the opposing hand can be inferred to be due to sharing of motor information and internal representations between hemisphere-hand systems in that specific sensorimotor domain (Kagerer 2015a).

It has been shown previously that visuomotor perturbation of the right hand causes interference in the left hand (Kagerer 2015b, 2016; Desrochers et al. 2020). However, preliminary evidence demonstrated that a dynamic perturbation does not produce substantial interference (Desrochers et al. 2017). While surprising, given that both perturbations theoretically require the updating of internal models, this finding is supported by research showing that learning of a dynamic perturbation in one hand neither interferes with nor facilitates learning of a separate dynamic perturbation in the opposing hand (Tcheang et al. 2007). Furthermore, different neural processes may be involved in the adaptation to visuomotor vs. dynamic perturbations, since one can be learned without simultaneously interfering with the other (Krakauer et al. 1999), though this does not necessarily hold when visuomotor and dynamic perturbation act with respect to the same kinematic variable (Tong et al. 2002). Several studies suggest that visual information dominates trajectory control over proprioceptive information (Judkins and Scheidt 2013; Sexton et al. 2019). For example, when participants are not provided with visual errors orthogonal to the direction of reaching, adaptation to a dynamic perturbation suffers, suggesting that visuomotor and dynamic motor information compete or regulate different phases of movement (Scheidt et al. 2005). The dominant sensory modality affecting a reach may also depend on the sensory information that informs the initial hand position, and thus, the motor plan (Sarlegna and Sainburg 2007). Few studies have investigated interplay between visual and dynamic sensory information in the context of bimanual coordination. Swinnen et al. (2001) demonstrated that adding force constraints to a bimanual spatial interference task did not result in additional interference. Diedrichsen (2007) showed that when participants control individual cursors with each hand and reach to separate targets, a force perturbation in the right hand did not influence the trajectory of the left hand.

Of course, dynamics are pertinent to motor learning and interference paradigms. Unimanual studies have shown an interaction between visuomotor and dynamic perturbations within the sensorimotor system. Franklin and colleagues (2012) demonstrated that while adapting to a dynamic perturbation, participants’ motor responses to a visuomotor perturbation are upregulated compared to when they are not adapting to a dynamic perturbation. This suggests that the presence of task relevant, novel dynamics may increase sensorimotor gain and upregulate responses to visuomotor perturbations while feed-forward internal models are being updated. In other words, adaptation to one type of sensorimotor information may increase responses to perturbations of the other type. Our lab has previously shown that reaching against resisting progressively increased forces yields progressively greater interference, particularly when one hand is undergoing sensorimotor adaptation (Brunfeldt et al. 2021). In that study, we showed that bimanual interference is affected by force demands and visuomotor adaptation using two experiments. In both, participants performed simultaneous reaching movements with each hand while varying levels of spring-like resistive force (0, 30, or 60 N/m) were applied to both hands. In the adaptive experiment, the right hand received a 40° clockwise visual rotation requiring sensorimotor adaptation, while the left hand had no visual feedback. In the non-adaptive experiment, the right hand reached to rotated target locations without any perturbation to visual feedback, again with the left hand under kinesthetic control only. Results showed that interference in the left hand increased with greater force demands and was significantly higher when the task required visuomotor adaptation, highlighting the role of feedback sensitivity and shared internal models in interlimb coordination. However, it is unknown whether, during a bimanual task, upregulating sensorimotor networks via simultaneous dynamic and visuomotor perturbations in one hemisphere-hand system will influence the contralateral hemisphere-hand system, manifesting increased interference compared to either perturbation alone.

In this experiment, we examined how visuomotor, dynamic, and combined visuomotor and dynamic perturbations of the right hand affected interference in the left, unperturbed hand. We hypothesized that if visuomotor and dynamic motor information are differentially shared between hemispheres, interference in the nondominant left hand due to perturbations of similar magnitude in the dominant right hand will be observed at disparate levels. Additionally, if the motor system responds to dynamic and visuomotor perturbations synergistically, as suggested by Franklin and colleagues (2012), interference in the left hand due to a simultaneous dynamic and visuomotor perturbation of the right hand would be greater than interference during dynamic or visuomotor perturbation alone.

## Methods

### Participants

We recruited sixty right-handed young adults, confirmed via the Edinburgh Handedness Inventory – Short form (Oldfield 1971), who were between 18 and 30 years old. Participants had no history of cognitive or neurological impairment, had not sustained a concussion in the past year, and had normal or corrected-to-normal vision. All participants provided informed consent, and all procedures were approved by the Michigan State University Institutional Review Board.

### Study design

**Fig. 1:**
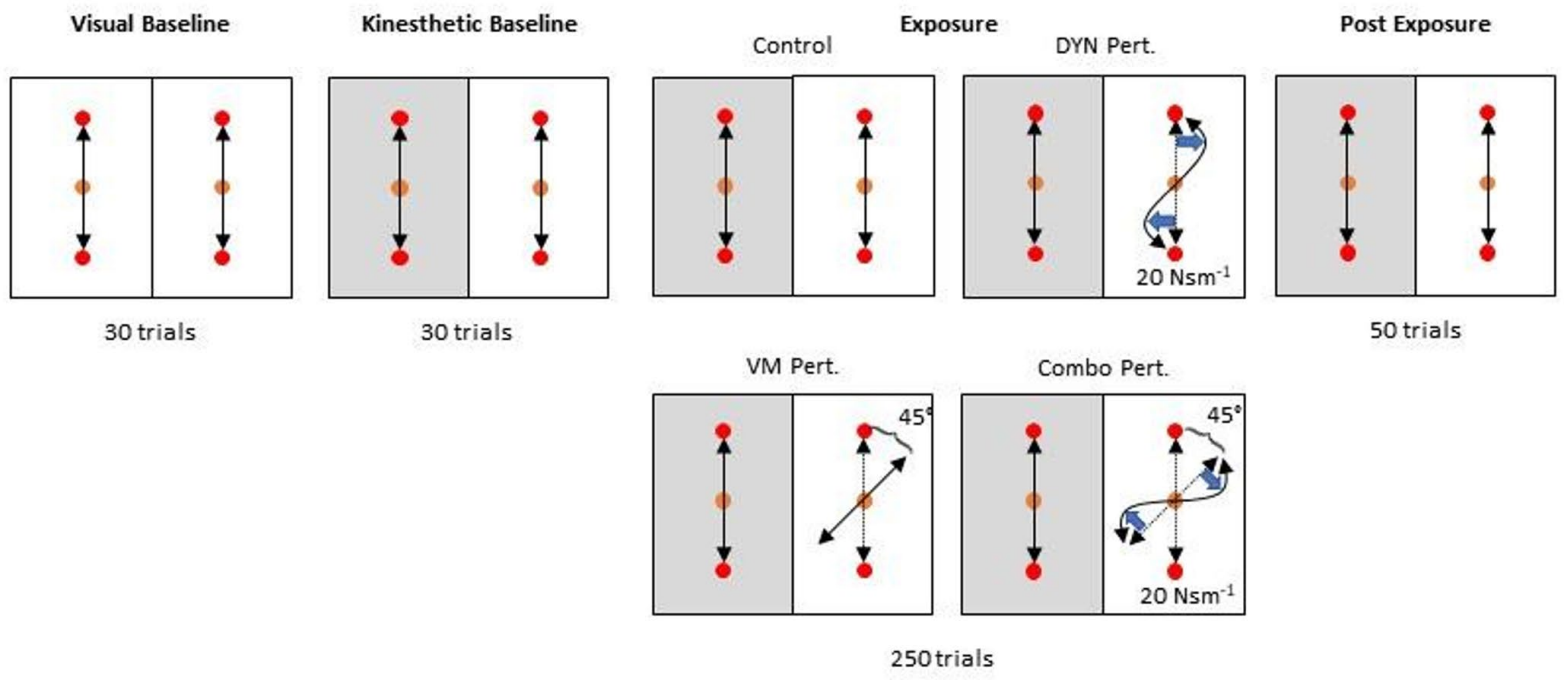
Boxes show the task design for the visual baseline, kinesthetic baseline, exposure, and post-exposure blocks. In the exposure blocks, the different groups received null (control), visuomotor, dynamic, or combined visuomotor and dynamic perturbations. Orange circles represent the home position, and red circles represent the targets. Shaded boxes indicate lack of visual feedback for the corresponding hand

**Fig. 2:**
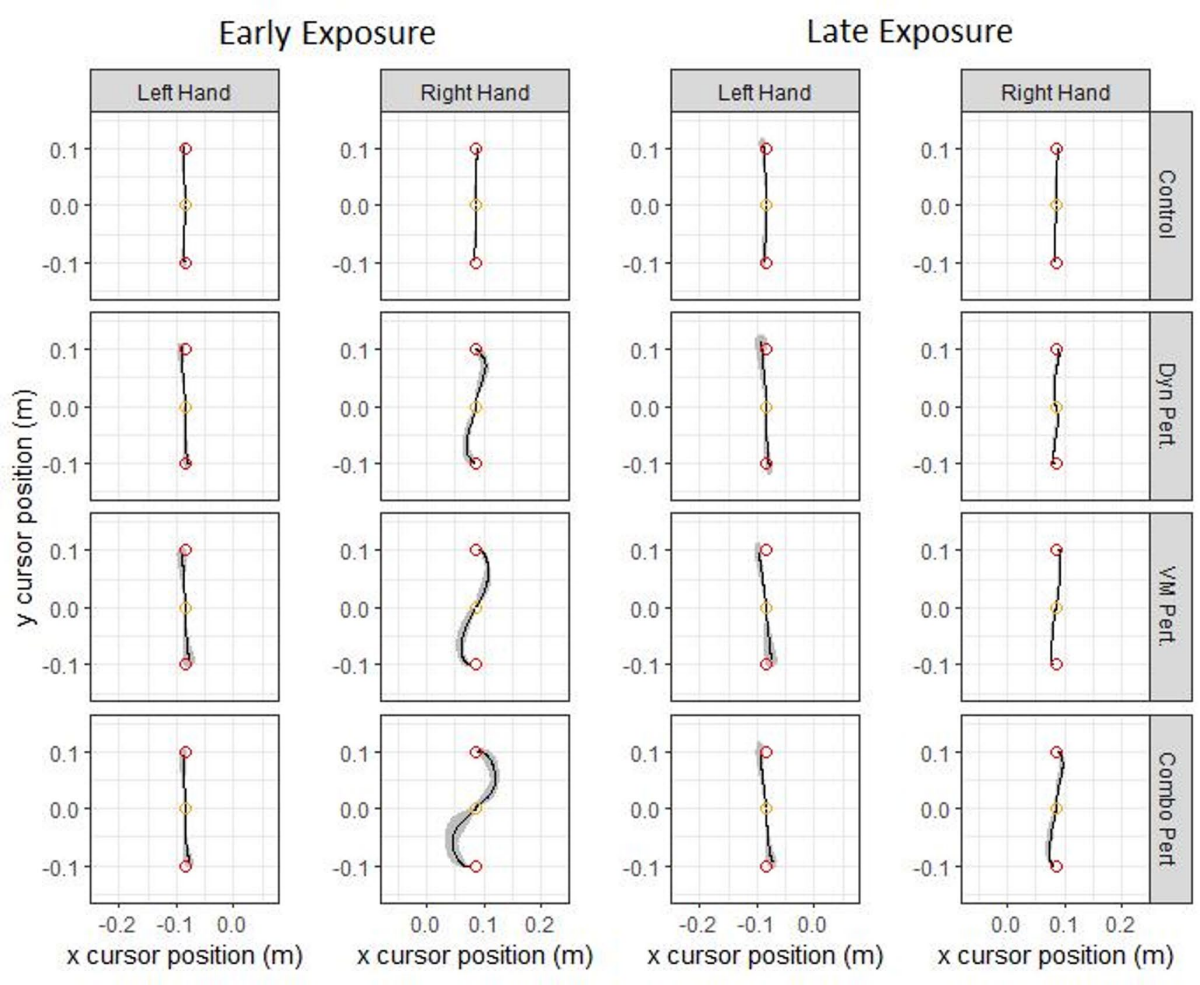
Hand paths for the left and right hands for each group across the Early- and Late-EXP blocks. Lines represent group means, clouds represent standard deviation of group hand paths. Colored circles represent the home position (orange) and targets (red)

Participants were randomly assigned to one of four groups (15 participants/group; Fig. 1): no perturbation, visuomotor perturbation, dynamic perturbation, or combined perturbation (simultaneous dynamic + visuomotor). Participants were cued to move two Kinarm End-Point Lab manipulanda (BKIN Technologies Ltd., Kingston, Ontario) simultaneously from two home positions to two targets located 10 cm directly forward (90°) or backward (270°) from the home positions. They were instructed to reach straight, fast, and accurately from the home position to the target. Targets were randomly presented, but both hands always moved in the same direction. After staying in the target position for 1500 ms, participants were cued to return to the home positions. Hand position was represented by a cursor on a screen that occluded vision of the hands. Participants started by performing unperturbed reaches during two blocks of 30 trials. In the first visual baseline (VBL) block, hand feedback was displayed for both hands. Then, in the kinesthetic baseline (KBL) block, visual feedback (cursor position) was removed for the left hand, requiring participants to rely on kinesthetic control. They were instructed to continue reaching to the target with their nonvisible hand, stopping when they estimated that their hand was in the target. Then, in the exposure (EXP) block of 250 trials, participants were exposed to the perturbation (Fig. 2). For the visuomotor perturbation group, the cursor representing the right hand was rotated 45° clockwise about the home position, such that participants needed to adapt their right-hand movement trajectory − 45° to hit the target. The dynamic perturbation group encountered a curl field force acting 90° perpendicular to the instantaneous velocity vector at 20 N per m/s of the reach velocity magnitude. The combined perturbation group received both perturbations simultaneously, such that the visual feedback was rotated 45° and a 20 N per m/s force perpendicular to the direction of instantaneous velocity was applied. The control group experienced no perturbation in the right hand. Finally, in the post-exposure (Post-EXP) block of 50 trials, the perturbations for the three experimental groups were removed. During the EXP and Post-EXP blocks, left hand visual feedback remained off, leaving the left hand susceptible to interference (see Fig. 1).

Since the dynamic perturbation was velocity dependent, and to ensure all participants reached with similar velocities, they received feedback on their movement times in form of a colored rectangle in the center of the screen: Red for times under 300 ms, indicating that the reach duration was too short; blue for times over 450 ms (reach duration too long), and green if the time was within a 300–450 ms window (ideal reach duration). Trials in which participants did not successfully hit the timing window were not excluded from analysis; subjects were simply reminded to continue trying to achieve movements with the proper velocity. Generally, participants were quite good at adhering to the timing constraints, particularly during unperturbed trials and at the end of the exposure period.

### Kinematic and kinetic outcome measures

Movement onset and offset were semi-automatically identified using custom written Matlab scripts (Mathworks, inc., Natick, MA) with an onset/offset algorithm (Teasdale et al. 1993) and were subsequently checked for accuracy by trained experimenters. To evaluate motor performance in the right hand and interference in the left hand, we assessed kinematic error via four primary outcome measures at each trial: (1) root mean square error (RMSE), a measure of movement straightness across the entirety of the reach, was calculated with reference to a straight target vector and normalized to movement length; (2) Initial directional error (IDE), a measure of feed-forward or predictive error of the motor plan, was computed at the moment of peak tangential velocity as the angle between a vector from the home position to the position of the cursor and a vector from the home position to the target; (3) Initial endpoint error (IEE), computed at end of the initial ballistic movement, or the moment where the primary reach ends (i.e., past peak velocity and before any subsequent corrective movements) as the angular deviation from the vector between the home position and the hand and the vector between the home position and the target. The end of the initial ballistic movement was defined as the first moment after peak tangential velocity where the hand’s velocity experienced a local minimum (i.e., the start of a secondary movement) or where the hand’s velocity crossed zero (i.e., a reversal of direction). IEE represents a moment from which on afferent sensory information is beginning to be integrated into the sensorimotor system, and online error correction is emerging as a result of feedback processes (Seidler 2006); (4) Final endpoint error (FEE), computed for the left hand as the lateral displacement between the hand position and the target at the moment when all movement had ceased in that hand. FEE occurred at a time in which the sensorimotor system had integrated all relevant feedback and corrected any perceived movement error.

We also applied a force channel to the left hand in 20% of trials that constrained movement to a straight-line path to the target, with a width of 0.25 mm. The robots linearly applied 20 N/cm as participants moved laterally into the walls; a 5 N lateral viscous force dampened possible harmonic oscillations between the two walls. The channels turned on gradually over a 1500 ms interval after the participants returned to and stopped in the home positions following the previous trial. When participants were informally asked after the experiment whether they noticed any manipulations in the left hand apart from loss of visual feedback, none of them reported noticing the force channel. Force sensors embedded in the Kinarm manipulanda endpoints measured the lateral force applied against the channel walls and served as an indicator of interference in the left hand. Each reach was interpolated to 1000 data points. The movement trajectories were averaged within early-EXP (first 30 trials of exposure) and late-EXP (last 30 trials of exposure). The force applied to the end-point handles was assessed at three timepoints during the reach: the average point of peak velocity (where IDE was calculated), the average point of the end of the initial ballistic movement (where IEE was calculated), and at the end of the movement (where FEE was calculated).

### Data processing and statistical analyses

Outcome measures for each dependent variable were baseline corrected: for the left hand, to the KBL block by subtracting the KBL block mean from each trial before averaging. For the right hand, the correction was done with respect to the VBL block of that hand. Kinematic and kinetic measures were evaluated in blocks that comprised the first and last 30 trials in the EXP period. A 2 (Block: Early, Late) x 4 (Group: Control, Visuomotor Perturbation, Dynamic Perturbation, Combined Perturbation) mixed-design ANOVA was used to assess adaptation in the right hand and interference in the left hand for each dependent measure. Violations of sphericity were adjusted with a Huynh-Feldt correction. Effect sizes were computed using generalized eta squared (*η*_*g*_^*2*^; (Bakeman 2005). Subsequent post-hoc analyses were performed by collapsing across non-significant independent variables, or by examining simple effects within blocks using one-way ANOVAs when significant main effects or interactions were present. Tukey HSD was used to determine differences between groups within each block. Statistical analyses were performed using custom written scripts in RStudio 1.2 software (RStudio Inc., Boston, MA) using the *afex*, *ez*, and base R packages.

## Results

### Kinematic measures

First, we wanted to confirm that the magnitudes of the visuomotor and dynamic perturbations of the right hand were comparable. Cursor trajectories are plotted in Fig. 2 across Early-EXP and Late-EXP for both hands. An independent samples t-test was used to evaluate the kinematic error in the right hand in the first 10 trials of EXP in the visuomotor and dynamic perturbation groups, before any substantial adaptation took place. No significant difference between the visuomotor and dynamic perturbation groups was found for RMSE (*t*(28) = −0.65, *p* = 0.52) or IEE (*t*(28) = 0.22, *p* = 0.83), though IDE was significant (*t*(28) = 7.98, *p* < 0.001). This showed that while the spatial magnitude of the perturbations was not equivalent at peak velocity, the net effect of the perturbation throughout the full movement trajectory was similar (as shown through IEE and RMSE). Thus, differences between the movements in the early, feed-forward components of the reach in the earliest moments of the EXP block would be expected, even when the overall effects were similar in magnitude.

Next, we evaluated participants’ adaptation to the respective perturbations. For RMSE across the exposure block, there were significant main effects of group (*F*(3, 56) = 85.16, *p* < 0.0001, *η*_*g*_^*2*^ = 0.77), and block (*F*(1, 56) = 463.50, *p* < 0.0001, *η*_*g*_^*2*^ = 0.68), with RMSE decreasing across exposure in the perturbation groups as participants adapted to the perturbations. A significant interaction was also present (*F*(3, 56) = 65.35, *p* < 0.0001, *η*_*g*_^*2*^ = 0.48), indicating adaptation in the perturbation groups while the control group’s error did not change across exposure. Similar patterns of results were also found for IDE and IEE (all *p* < 0.0001). As such, subsequent analyses of right-hand adaptation focused on RMSE as a measure of error across the full reach. Within each block of RMSE, one-way ANOVAs revealed significant main effects of group at early-EXP (*F*(3, 56) = 84.44, *p* < 0.0001, *η*_*g*_^*2*^ = 0.82) and late-EXP (*F*(3, 56) = 54.36, *p* < 0.01, *η*_*g*_^*2*^ = 0.74). Tukey HSD tests within each block, with family-wise adjustment for four estimates, showed that all three perturbation groups had significantly greater RMSE than controls at early EXP (all *p* < 0.01). The combined perturbation group showed significantly different RMSE than the dynamic and visuomotor perturbation groups (*p* < 0.0001), while the dynamic and visuomotor perturbation groups did not differ from one another. At late EXP, all groups again showed significantly greater RMSE than the control group (*p* < 0.01). Additionally, all perturbation groups were significantly different from one another (*p* ≤ 0.01), with the greatest RMSE in the combined perturbation group, followed by the dynamic perturbation, visuomotor, and control groups.

**Fig. 3:**
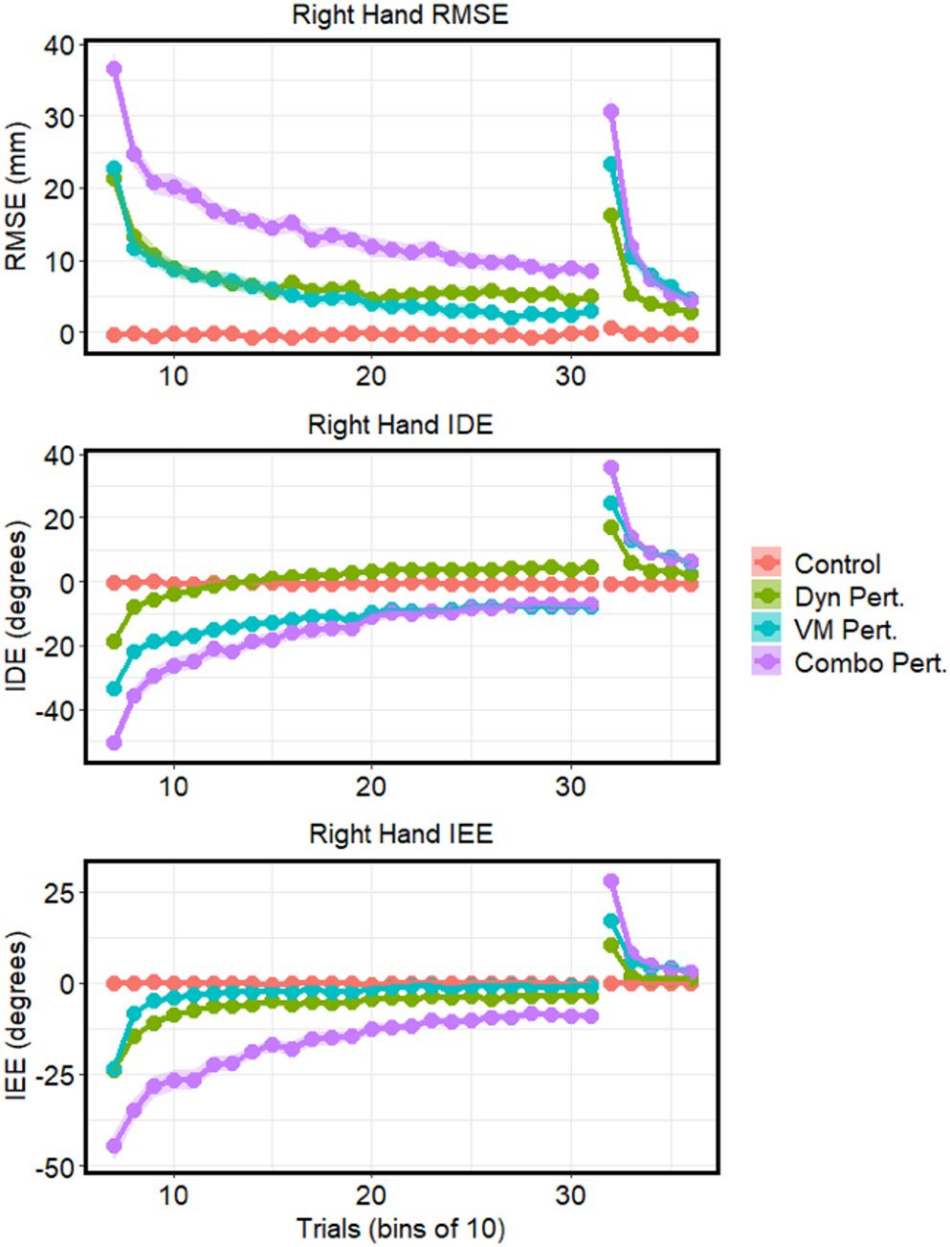
Right hand RMSE, IDE, and IEE across the exposure and post-exposure blocks. Points represent means of 10 consecutive trials. Clouds denote standard error. For IDE and IEE, angles in the first and third quadrants were negative (origin at home position)

We then investigated aftereffects by performing a 2 (Late EXP vs. first 30 trials post-EXP) x 4 (Group) mixed-design ANOVA. If participants had adapted to the perturbation, a subsequent increase in error upon removal of the perturbation was expected. Unsurprisingly, we found a main effect of group, showing that RMSE was significantly larger in the perturbation groups following removal of the perturbation (*F*(3, 56) = 104.70, *p* < 0.0001, *η*_*g*_^*2*^ = 0.74), and a main effect of block, with greater RMSE in the post-EXP block (*F*(1, 56) = 134.37, *p* < 0.0001, *η*_*g*_^*2*^ = 0.54). A significant interaction was also present (*F*(3, 56) = 22.89, *p* < 0.0001, *η*_*g*_^*2*^ = 0.37). A similar pattern of findings was observed for both IDE and IEE (all *p* < 0.01). Thus, remaining analyses again focused on RMSE. Analysis of simple effects at post-EXP revealed a significant main effect of group (*F*(3, 56) = 68.38, *p* < 0.0001, *η*_*g*_^*2*^ = 0.79), and subsequent Tukey HSD post-hoc tests showed that all groups were significantly different from each other (*p* = 0.001), with the exception of the visuomotor and combined perturbation groups, which did not differ (*p* = 0.12). The combined perturbation group showed the greatest aftereffects, followed by the visuomotor perturbation group and dynamic perturbation group. These demonstrate that all perturbation groups were able to successfully adapt to the perturbation and had significant aftereffects once the perturbation was removed (Fig. 3).

**Fig. 4:**
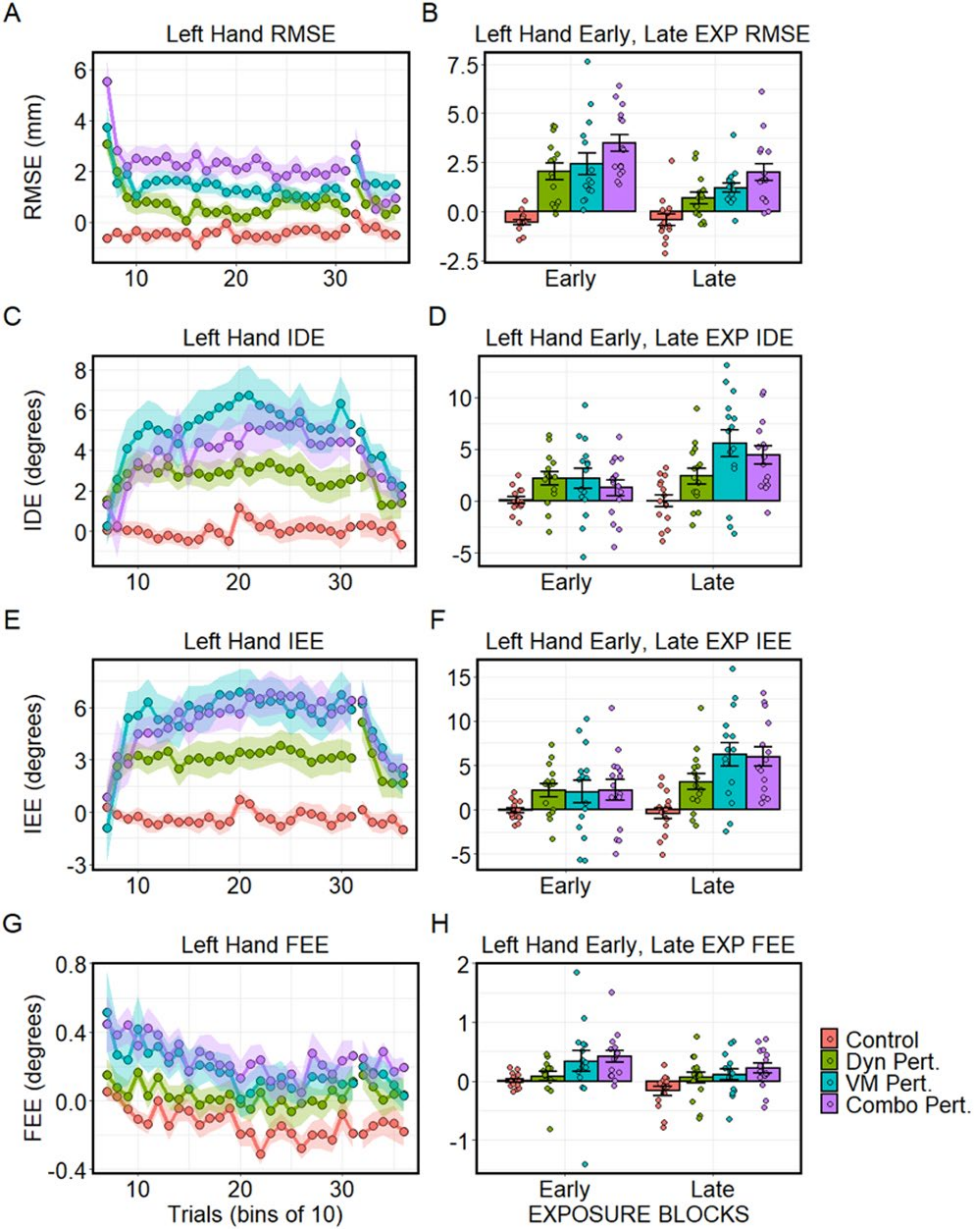
Left hand kinematic dependent variables. **A** RMSE, **B** IDE, **C** IEE, & **D** FEE. Means are baseline corrected to the KBL block. For IDE and IEE, angles in the first and third quadrants were negative (origin at home position); thus, interference manifested as a counterclockwise bias in the movement direction. Left panels depict each respective measure in bins of 10 trials each across the exposure (trial bins 7–31) and post-exposure (trial bins 32–36) blocks. Clouds represent standard error (SE). Bars in panels B, D, F, H depict mean ± SE, while points represent individuals subjects for each respective measure at early-EXP (first 30 trials of EXP) and late-EXP (last 30 trials of EXP) for each group

After establishing the adaptation characteristics of the groups’ perturbed right hands, we investigated the reaching behavior of the left, interfered-with hand. For RMSE, the overall measure of left-hand reaching error, a mixed-design ANOVA revealed significant main effects of group (*F*(3, 56) = 18.33, *p* < 0.0001, *η*_*g*_^*2*^ = 0.42) and block (*F*(1, 56) = 25.09, *p* < 0.0001, *η*_*g*_^*2*^ = 0.11), with RMSE decreasing across the exposure period (Fig. 4A & B), and a significant Group x Block interaction (*F*(3, 56) = 3.62, *p* = 0.02, *η*_*g*_^*2*^ = 0.05), showing that while interference in left hand changed across exposure for the perturbation groups, movement error remained relatively unchanged for the control group. Examination of the simple effects in both early- and late-EXP showed significant group main effects (all *p* < 0.0001, *η*_*g*_^*2*^ > 0.30). In early-EXP, Tukey HSD showed that all perturbation groups were significantly different from the controls (all *p* < 0.001). However, after correcting for multiple comparisons, RMSE did not significantly differ between the perturbation groups, though there was a trend for a difference between the combined perturbation and dynamic perturbation groups (*p* = 0.08). In late-EXP, the combined and visuomotor perturbation groups showed significantly higher RMSE than the controls (*p* < 0.01), whereas the dynamic perturbation group did not. Between perturbation groups, the difference between the combined and dynamic perturbation groups was significant (*p* = 0.03). Taken together, all three perturbation groups showed more interference than controls, with progressively more interference across the dynamic, visuomotor, and combined perturbations.

For IDE in the left hand, indicative of feed-forward reaching error, the mixed-design ANOVA showed significant main effects of group (*F*(3, 56) = 4.82, *p* < 0.01, *η*_*g*_^*2*^ = 0.17) and block (*F*(1, 56) = 22.71, *p* < 0.0001, *η*_*g*_^*2*^ = 0.07). Interference increased over the course of exposure, mostly driven by the visuomotor and combined perturbation groups (Fig. 4C & D). The interaction term was also significant (*F*(3, 56) = 6.90, *p* < 0.001, *η*_*g*_^*2*^ = 0.06). Examination of simple effects showed that group differences were not statistically significant in early-EXP (*F*(3, 56) = 1.98, *p* = 0.13, *η*_*g*_^*2*^ = 0.10), but achieved significance in late-EXP (*F*(3, 56) = 7.14, *p* < 0.001, *η*_*g*_^*2*^ = 0.28). In late-EXP, Tukey HSD showed that the visuomotor group had significantly higher IDE than the controls (*p* < 0.001), and marginally greater IDE than the dynamic perturbation group (*p* = 0.08). Additionally, the combined perturbation group had significantly higher IDE than controls (*p* < 0.01). All other contrasts were not significant after correcting for multiple comparisons. Taken together, these results show that interference due to feed-forward control processes was greatest for the visuomotor and combined perturbation conditions, especially at the end of exposure.

For left-hand IEE, measuring end-point error at the end of the initial ballistic movement, the mixed-design ANOVA revealed significant main effects of group (*F*(3, 56) = 5.85, *p* < 0.01, *η*_*g*_^*2*^ = 0.19) and block (*F*(1, 56) = 19.16, *p* < 0.0001, *η*_*g*_^*2*^ = 0.08), with IEE increasing over the course of the exposure block (Fig. 4E & F). The interaction term was also significant (*F*(3, 56) = 5.07, *p* < 0.01, *η*_*g*_^*2*^ = 0.06). Within each block, main effects of group were not significant in early-EXP (*F*(3, 56) = 1.39, *p* = 0.25, *η*_*g*_^*2*^ = 0.07), but achieved significance in late-EXP (*F*(3, 56) = 9.35, *p* < 0.0001, *η*_*g*_^*2*^ = 0.33). In late-EXP, the visuomotor perturbation (*p* < 0.001) and combined perturbation (*p* < 0.01) groups developed significantly higher IEE in their left hand than controls, while in the dynamic group it was marginally different from the controls (*p* = 0.07). Differences between the perturbation groups were not significant after correcting for multiple comparisons. This shows that, at the end of the initial ballistic movement, the visuomotor and combined perturbation groups produced the most interference, particularly at the end of the exposure period, while interference due to the dynamic perturbation of the right hand was lower.

**Fig. 5:**
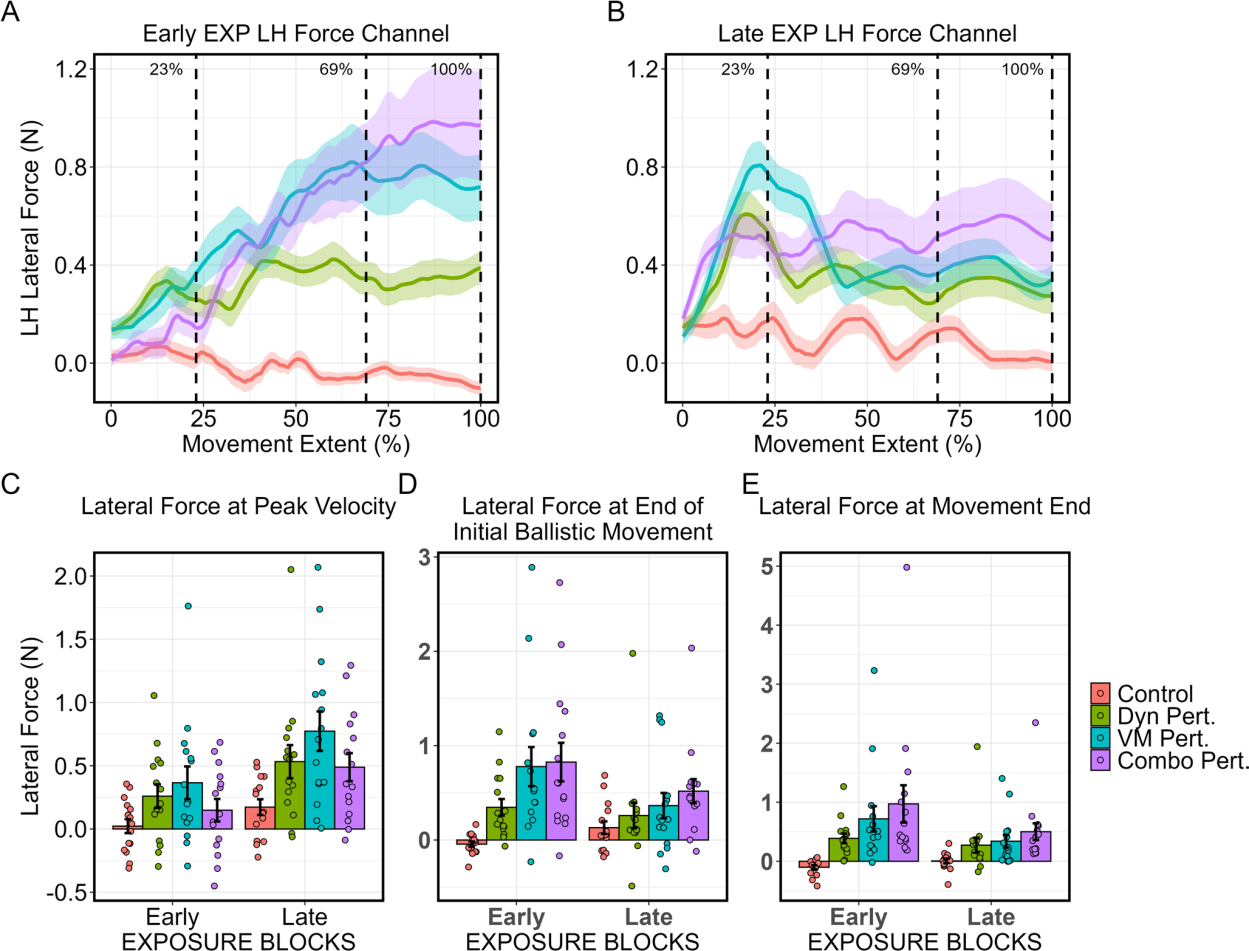
Left hand kinetic results during force channel trials. Top row shows the force applied against the force channel throughout the movement in early-EXP (**A**), and late-EXP (**B**). Bars in C, D, and E depict group mean ± SE, while points represent individual subjects at the average point of peak velocity (**C**), end of the initial ballistic movement (**D**), and at the end of the movement (**E**), corresponding 23%, 69%, and 100% of the way through the movement, respectively. Please note that y-axis of C, D, and E were allowed to vary across plots to depict all datapoints

For FEE, measuring the lateral displacement of the left hand from the target once all movement had ceased and incorporating feedback driven corrections at the movement endpoint, the mixed-design ANOVA revealed main effects of group (*F*(3, 56) = 5.08, *p* < 0.01, *η*_*g*_^*2*^ = 0.14) and block (*F*(1, 56) = 6.27, *p* = 0.02, *η*_*g*_^*2*^ = 0.04), with FEE decreasing across exposure (Fig. 5G & H). The interaction term was not significant. In early-EXP, the main effect of group was significant (*F*(3, 56) = 3.15, *p* = 0.03, *η*_*g*_^*2*^ = 0.14), and remained significant at late-EXP (*F*(3, 56) = 3.46, *p* = 0.02, *η*_*g*_^*2*^ = 0.16). Tukey HSD revealed that that the combined perturbation group had greater deviation than the control group in early-EXP (*p* = 0.06) and in late-EXP (*p* = 0.01). These results show that at the end of movement, the combined perturbation group exhibited the greatest amount of interference, but not over and above that of the other two perturbation groups.

### Kinetic measures

A virtual force channel interspersed pseudorandomly in 20% of trials constrained movement of the left hand to a straight line between home position and targets. Across participants and trials, the average point of peak velocity was at 23% of the whole movement, while the average point of the end of the initial ballistic movement was at 69% of the movement. As such, lateral force applied against the channel wall by the left hand was evaluated between groups at early- and late-EXP at these points, as well as at the end of the movement (Fig. 5 A and B).

At average peak velocity (Fig. 5 C), early on in the reach, the mixed-design ANOVA revealed significant main effects of both group (*F*(3, 56) = 4.55, *p* < 0.01, *η*_*g*_^*2*^ = 0.15) and block (*F*(1, 56) = 25.58, *p* < 0.0001, *η*_*g*_^*2*^ = 0.12), with the lateral force increasing from early- to late-EXP; the interaction term was not significant. Examination of the simple effects in each phase showed a marginal difference between groups in early-EXP: *F*(3, 56) = 2.38, *p* = 0.08, *η*_*g*_^*2*^ = 0.11) and a significant group difference in late-EXP (*F*(3, 56) = 4.22, *p* < 0.01, *η*_*g*_^*2*^ = 0.18). In early- and late-EXP, post-hoc tests showed that the visuomotor perturbation group produced greater lateral force against the channel walls than controls (Early-EXP: *p* = 0.06; Late-EXP: *p* < 0.01). No other contrasts were significant.

At the end of the initial ballistic movement (Fig. 5D), ANOVA revealed significant main effects of group (*F*(3, 56) = 5.10, *p* < 0.01, *η*_*g*_^*2*^ = 0.19) and block (*F*(1, 56) = 8.19, *p* < 0.01, *η*_*g*_^*2*^ = 0.02), driven by a decrease in lateral force generated by the visuomotor and combined perturbation groups across the exposure period. The interaction term was also significant (*F*(3, 56) = 5.53, *p* < 0.01, *η*_*g*_^*2*^ = 0.05). Examination of the simple effects showed a significant group main effect at early-EXP (*F*(3, 56) = 7.17, *p* < 0.001, *η*_*g*_^*2*^ = 0.28), with the combined perturbation (*p* < 0.01) and visuomotor perturbation (*p* < 0.01) generating greater lateral force than the control group. At late-EXP, the group effect was not significant, though visual inspection of the force produced by the left hand showed the dynamic, visuomotor, and combined perturbation groups each producing respectively more force against the channel.

At the end of the movement (Fig. 5E), ANOVA showed significant main effects of group (*F*(3, 56) = 5.19, *p* < 0.01, *η*_*g*_^*2*^ = 0.19) and block (*F*(1, 56) = 11.95, *p* < 0.01, *η*_*g*_^*2*^ = 0.03), and a significant interaction (*F*(3, 56) = 4.46, *p* < 0.01, *η*_*g*_^*2*^ = 0.04), again driven by decreasing lateral force generated by the combined and visuomotor perturbation groups across exposure. Examination of simple effects showed significant group differences in early-EXP (*F*(3, 56) = 5.61, *p* < 0.01, *η*_*g*_^*2*^ = 0.23) and late-EXP (*F*(3, 56) = 3.45, *p* = 0.02, *η*_*g*_^*2*^ = 0.16). The combined perturbation group generated significantly more force than controls in both early-EXP (*p* < 0.01) and late-EXP (*p* = 0.01), while the visuomotor group generated greater force than controls in early-EXP only (*p* = 0.02).

Taken together, these results show that before participants adapted, the visuomotor and combined perturbation groups produced the greatest amount of force against the channel, particularly at the end of the movement. At the end of the exposure period, once participants had adapted, the visuomotor perturbation group produced the greatest amount of force against the channel early in the movement, while the combined perturbation group produced the greatest force at the end of the movement.

## Discussion

This study examined how adaptation of one hand to either visuomotor or dynamic perturbations affects the contralateral hand, manifesting as interference. First, based on prior experimental data (Desrochers et al. 2017) we predicted that interference in the left hand would be greater when the right hand adapted to a visuomotor rather than a dynamic perturbation. This was confirmed in our results; across several dependent measures, visuomotor perturbation resulted consistently in greater interference than dynamic perturbation, and it was particularly pronounced in measures that evaluated feed-forward and early feedback driven control (IDE and IEE, respectively), and later in the exposure phase, when participants’ right hand had adapted to the perturbations. The effect was less pronounced in variables that incorporated feedback driven processes (FEE and RMSE). This may reflect the dominance of visual information in controlling movement trajectories to visual targets (Scheidt et al. 2005; Judkins and Scheidt 2013; Sexton et al. 2019).

Secondly, our hypothesis that interference would be greater when participants were exposed to both perturbations simultaneously, was only partially supported by our results. In measures of feed-forward driven processes, which assessed movement parameters prior to the integration of reaching feedback (IDE and, to a lesser extent, IEE), the group that received both perturbations in the right hand tended to show roughly equivalent (and sometimes, even slightly less) interference in the left hand compared to the group that received the visuomotor perturbation alone. This occurred despite the combined perturbation being (at peak velocity) larger in magnitude than either the visuomotor or dynamic perturbation. At the same time, in measures that assessed feedback driven processes later in the movement such as RMSE and FEE, it was the combined perturbation group that showed the greatest interference. This pattern was also evident from the kinetic measures – at the end of the exposure period, early on in a given movement (i.e., at peak velocity), it was the visuomotor perturbation that showed the greatest interference, and late in the movement (i.e., at the end of the initial ballistic movement and end of the reach), it was the combined perturbation that resulted in greater interference. Force profiles in the force channel catch trials showed substantial change from early to late adaptation (see Fig. 6), especially at points dominated by feed-forward processes, possibly reflecting explicit processes and limb impedance in early adaptation versus updated internal models in late adaptation.

These results connect to previous findings that the sensorimotor system can selectively upregulate its sensitivity to certain types of feedback during learning. For example, participants respond more strongly to a rapid visuomotor perturbation when they are adapting to a dynamic perturbation, compared to performing normal reaches (Franklin et al. 2012). These responses can also be selectively tuned to different factors affecting the reach, showing that the nervous system can not only learn a specific perturbation, but also modulate secondary mechanisms to adapt to the environment at large (Dimitriou et al. 2011; Franklin et al. 2014, 2017). Selectively upregulating sensitivity to certain types of sensorimotor information is thought to allow the sensorimotor system to rapidly correct for further perturbation during environmental uncertainty. Such uncertainty can arise from internal sources such as feedback delays or increased sensorimotor noise (Churchland et al. 2006; Faisal et al. 2008; van Beers 2009), or from external sources such as limited feedback or an unpredictable environment (Cluff and Scott 2013; Nashed et al. 2014). Importantly, the ability to scale responses in a context-dependent fashion is a key prediction of optimal feedback control theory (OFTC; (Todorov and Jordan 2002; Scott 2004; Todorov 2005; Diedrichsen et al. 2010; Scott et al. 2015; Franklin et al. 2017)

Despite this compelling evidence of gain scaling in unimanual studies, this study suggests that sensorimotor upregulation may not be shared with the contralateral hemisphere-hand system in our task, particularly in the case of dynamic adaptation. If gain scaling parameters are shared, e.g., via neural crosstalk, the presence of a dynamic perturbation with a visuomotor perturbation in the right hand should have amplified the effects of interference across effectors. Research suggests feedback gain responses in each effector can be independently controlled by the sensorimotor system during bimanual movements. Using a task where cursors shifted after a visual occlusion bar in one hand or in both hands during bimanual reaches, Brouwer and colleagues (2017) found not only a decrease in feedback gains during bimanual movements but also that the sensorimotor system could independently control responses in each hand. As such, each hand could modify its gain response with respect to its own goal. This response is in line with OFCT, which predicts that the sensorimotor system will constrain variability and motor responses if they affect the overall task goal (Diedrichsen 2007).

In the current experiment, interference in the combined perturbation group may not have increased beyond that of the visuomotor perturbation alone because the left hand was not required to interact with a dynamic perturbation and was therefore not susceptible to the effects of the perturbation in the right hand. This is likely due to dynamic adaptation occurring in independent, intrinsic frames of reference specific to the adapting hand (Shadmehr and Mussa-Ivaldi 1994). It is important to note that, since we only assessed dominant-to-nondominant hand interference, we could not capture potential functional control asymmetries previously described in unimanual (Sainburg 2002), bimanual (Kagerer 2015b, 2016) tasks, or bimanual proprioceptive matching tasks (Goble et al. 2006). Overall, our results suggest that feedback gains within each hemisphere-hand system can be independently regulated in parallel with one another. Further, it suggests that these upregulated feedback-gains in one hemisphere-hand system are not necessarily communicated between hemispheres via neural crosstalk.

This study also raises interesting questions as to why visuomotor perturbations produce greater interference than dynamic perturbations during bimanual movements. One could argue that a simple answer may lie in the fact that in the visuomotor perturbation task, the movements of the hands are inherently asymmetrical, as the right hand must change its trajectory to hit the target. In this context it is important to note that if interference was strictly due to asymmetrical reaches alone, it would be present at its maximal magnitude as soon as the task required the participants to reach asymmetrically, especially in measures taken late in the reach (IEE, FEE). FEE and IEE did not show robust differences between groups in early-EXP. Moreover, force exerted against the force channel in the left hand was lower in the early part of the reach in early-EXP, but at its greatest magnitude in late-EXP, potentially indicating an adapted internal response. Finally, the time course of interference in our task, which increases gradually over the course of exposure, lends support to interference being enhanced as a result of adaptive processes. Yet it is important to acknowledge that the increase in interference does coincide with adaptation in the right hand resulting in more consistently straight, directionally asymmetrical reaches towards the targets. Most participants anecdotally reported that they were unaware that their reaches were asymmetrical by the end of the exposure period in the visuomotor condition. Instead, they perceived their movements as being symmetrical – yet interference remained present towards the end of exposure. While movement asymmetry might account for some interference effects, these data show that adaptive processes contribute to crosstalk between hemisphere hand systems. Thus, the differences in interference between directional and adaptation-driven perturbation types may be at least in part, driven by neural processes tied to adapting to the visuomotor perturbation itself (see also Brunfeldt et al. 2021). More work is needed to tease apart the contributions of adaptive processes vs. reaching asymmetry in interference between the hands.

As such, the neural circuitry involved in adapting to these perturbations may play an integral role in the observed differences in interference. Research suggests that adaptation to visuomotor and dynamic perturbations may recruit slightly different neural substrates, particularly in the cerebellum (Rabe et al. 2009; Donchin et al. 2011). Additionally, visuomotor control also requires a complex premotor-parietal network responsible for localizing targets in space and planning movement trajectories, in addition to contribution of M1 (Kurata 1994; Desmurget et al. 1999; Tanaka et al. 2009; Mutha et al. 2011; Werner et al. 2014; Culham 2015; Manuweera et al. 2018). Critically, visuospatial information is also projected to both hemispheres, requiring posterior parietal cortices of both hemispheres to successfully plan spatial movements (Goodale and Milner 1992), and the parietal cortices are implicated in contributing to bimanual interference (Iester et al. 2025). As such, is not surprising that severing portions of the corpus callosum that connect posterior parietal cortex preferentially abolishes spatial interference (Eliassen et al. 1999). Further, the left parietal lobe is particularly involved in visuomotor adaptation (Mutha et al. 2011); this may contribute to the elevated role of visuomotor feedback on interference demonstrated in this study.

These perturbations may also be functionally distinct. Visuomotor and dynamic perturbations do not interfere with one another (Krakauer et al. 1999; Tcheang et al. 2007), and likely use different reference frames (Shadmehr and Mussa-Ivaldi 1994; Flanagan and Rao 1995). Evidence also shows that neuromuscular adaptation to visuomotor rotations is specific to the subspace in which the perturbation takes place (Severini and Zych 2020). Individuals who adapt well to velocity dependent dynamic perturbations do not necessarily perform well adapting to visuomotor rotations (Moore and Cluff 2021). Individuals with extensive experience in the complex sensorimotor task of videogaming show enhanced adaptation to visuomotor perturbations but not dynamic perturbations (Kunavar et al. 2025). However, evidence suggests that multiple reference frames may be used depending on the context of the movement (Berniker et al. 2013; Parmar et al. 2015) and that perturbations do indeed interact when applied to the same motor variable (Tong et al. 2002). As such, it is possible that visuomotor perturbations affect reference frames that are shared between effectors, whereas dynamic perturbations use reference frames that are isolated in a single effector.

A limitation of our study might be that we used a velocity dependent curl field as dynamic perturbation, as this type of force field tends to be used more often in the literature. However, a position-dependent force field (e.g., Sing et al. 2009) may have allowed for better comparison between the dynamic and visuomotor perturbations, as the magnitude of the perturbation would be better matched over the course of the reach. More recent evidence does show that congruent visual and dynamic perturbation accelerates adaptation (Franklin et al. 2023). Nevertheless, if adapting to one type of sensorimotor perturbation upregulates sensorimotor feedback gains and increases sensitivity to a different sensorimotor perturbation (Franklin et al. 2012, 2017), the velocity dependent force field used in the present study should still have produced upregulation of sensorimotor feedback gains in the combined perturbation group.

In summary, this study has shown that visuomotor and dynamic perturbations with similar magnitude show different levels of left-hand interference, with the visuomotor perturbation eliciting greater interference, particularly in feed-forward measures at the end of an adaptation period. Also, simultaneously adapting to visuomotor and dynamic perturbations in one hand does not increase interference in the opposing hand beyond that of a visuomotor perturbation alone. Understanding how sensorimotor information is shared between hemispheres may not only inform us about these processes in healthy individuals but might also help improving approaches to facilitating recovery of function, e.g., in stroke patients. Despite heterogeneous findings in this population, and dearth of hypothesis-guided studies using bimanual interventions, several studies studies suggest that bimanual training approaches may increase training benefits, e.g., by guiding the more impaired through the less impaired limb when performing movements (see Rose and Winstein 2004; Sainburg et al. 2013). Exploiting interlimb interferences could be another approach to facilitate recovery of function in some of these patients.

## Data Availability

Data and analysis code can be found in PCD’s GitHub repository at https://github.com/philcd89.

## Abbreviations

ANOVA: Analysis of Variance
Combo Pert.: Combined Perturbation
DYN Pert.: Dynamic Perturbation
EXP: Exposure
FEE: Final Endpoint Error
IDE: Initial Directional Error
IEE: Initial Endpoint Error
KBL: Kinesthetic baseline
OFCT: Optimal Feedback Control Theory
Post-EXP: Post-exposure
RMSE: Root mean square error
VBL: Visual baseline
VM Pert.: Visuomotor perturbation
